# Correction: Starkute et al. Characteristics of Unripened Cow Milk Curd Cheese Enriched with Raspberry (*Rubus idaeus*), Blueberry (*Vaccinium myrtillus*) and Elderberry (*Sambucus nigra*) Industry By-Products. *Foods* 2023, *12*, 2860

**DOI:** 10.3390/foods14101831

**Published:** 2025-05-21

**Authors:** Vytaute Starkute, Justina Lukseviciute, Dovile Klupsaite, Ernestas Mockus, Jolita Klementaviciute, João Miguel Rocha, Fatih Özogul, Modestas Ruzauskas, Pranas Viskelis, Elena Bartkiene

**Affiliations:** 1 Department of Food Safety and Quality, Faculty of Veterinary, Lithuanian University of Health Sciences, Tilzes Str. 18, LT-47181 Kaunas, Lithuania; vytaute.starkute@lsmu.lt (V.S.); justina.lukseviciute@lsmu.lt (J.L.); 2 Faculty of Animal Sciences, Institute of Animal Rearing Technologies, Lithuanian University of Health Sciences, Tilzes Str. 18, LT-47181 Kaunas, Lithuania; dovile.klupsaite@lsmu.lt (D.K.); ernestas.mockus@lsmu.lt (E.M.); jolita.klementaviciute@lsmu.lt (J.K.); 3 Universidade Católica Portuguesa, CBQF—Centro de Biotecnologia e Química Fina—Laboratório Associado, Escola Superior de Biotecnologia, Rua Diogo Botelho 1327, 4169-005 Porto, Portugal; jmfrocha@fc.up.pt; 4 Laboratory for Process Engineering, Environment, Biotechnology and Energy (LEPABE), Faculty of Engineering, University of Porto (FEUP), Rua Dr. Roberto Frias, s/n, 4200-465 Porto, Portugal; 5 Associate Laboratory in Chemical Engineering (ALiCE), Faculty of Engineering, University of Porto, Rua Dr. Roberto Frias, s/n, 4200-465 Porto, Portugal; 6 Department of Seafood Processing Technology, Faculty of Fisheries, Cukurova University, Balcali, Adana 01330, Turkey; fozogul@cu.edu.tr; 7 Biotechnology Research and Application Center, Cukurova University, Balcali, Adana 01330, Turkey; 8 Department of Anatomy and Physiology, Faculty of Veterinary, Lithuanian University of Health Sciences, Tilzes Str. 18, LT-47181 Kaunas, Lithuania; modestas.ruzauskas@lsmu.lt; 9 Faculty of Veterinary, Institute of Microbiology and Virology, Lithuanian University of Health Sciences, Tilzes Str. 18, LT-47181 Kaunas, Lithuania; 10 Lithuanian Research Centre for Agriculture and Forestry, Institute of Horticulture, Kauno Str. 30, LT-54333 Babtai, Lithuania; pranas.viskelis@lammc.lt

With this Correction the journal’s Editorial Office and Editorial Board are jointly issuing a resolution to the Expression of Concern [1] on the article “Characteristics of Unripened Cow Milk Curd Cheese Enriched with Raspberry (*Rubus idaeus*), Blueberry (*Vaccinium myrtillus*) and Elderberry (*Sambucus nigra*) Industry By-Products. *Foods* **2023**, *12*, 2860” by Starkute et al. [2]. This correction, details listed below, addresses the concerns raised in the above-mentioned Expression of Concern and is complimented by the addition of the study’s raw materials to the published article (accessible via https://www.mdpi.com/2304-8158/13/14/2250, accessed on 8 April 2025).

## Error in Figure

In the original publication [2], there was a technical mistake in Figure 3 as published. The corrected Figure 3 appears below.




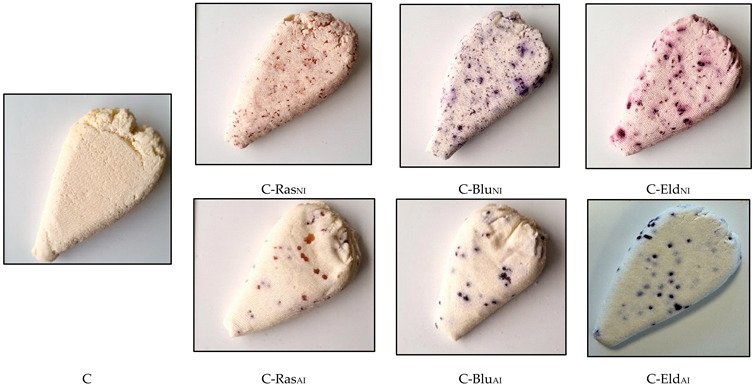




## Text Correction

In Section 3.1.2. Antioxidant Characteristics, Color Coordinates (L*, a* and b*), pH and Acidity (TTA) Parameters of Berry Industrial By-Products (BIBs), paragraph 2, the following corrections should be included:

“Essentially, the BIBs, i.e., those obtained after juice manufacturing, consist of pulp, peels and seeds [49] and contain considerable amounts of anthocyanins, especially in the dark color berries. It was reported that raspberry, blueberry and elderberry BIBs are rich in these compounds and possess high antioxidant activity [50–55]. **Notably, the DPPH radical scavenging activity depends on the berry variety, region of cultivation and the chosen extraction and drying method. Četojević-Simin et al. reported that raspberry cultivar ‘Willamette’ BIB extracts possess 43.7 ± 2.02 mg GAE/g of total phenolic content [56].** Our results showed that the highest TPC content is obtained in elderberry BIBs. **Tánska et al. reported that the TPC content in elderberry pomace is 13.86 ± 0.22 g/100 g [57]**. In comparison with blueberries, elderberries have higher anthocyanin and phenolic compound contents [58]. Moreover, antioxidant activity can be a result of synergic interactions among antioxidant compounds [59].”

To support the results, the raw data file has been provided as Supplementary File S6 and appended to the Supplementary Materials section, as per the Journal’s request.

In section Supplementary Materials the following information should be included:

**Supplementary Materials:** The following supporting information can be downloaded at: https://www.mdpi.com/article/10.3390/foods12152860/s1, Supplementary File S1: Method of antioxidant properties; Supplementary File S2: Method for fatty acids; Supplementary File S3: Method for volatile compounds profile; Supplementary File S4: Method for biogenic amines content; Supplementary File S5: Method for evaluation of overall acceptability and induced emotions for consumers; Supplementary File S6: Raw data file.

The authors state that the scientific conclusions are unaffected. This correction was approved by the Academic Editor. The original publication has also been updated.
