# Supplementary material for: Characteristics of Unripened Cow Milk Curd Cheese Enriched with Raspberry (Rubus idaeus), Blueberry (Vaccinium myrtillus) and Elderberry (Sambucus nigra) Industry By-Products
Source: Foods. 2023 Jul 27;12(15):2860. doi: 10.3390/foods12152860 (PMC10417324; doi:10.3390/foods12152860)
Supplement: Supplementary file 1 [file foods-12-02860-s001.zip › foods-2507967-supplementary.pdf]

Supplementary File S1_Method for antioxidant properties

Determination of total phenolic compounds (TPC) and 2,2-diphenyl-1-picrylhydrazyl (DPPH·)-radical scavenging activity

The TPC content of the BIBs was determined by a spectrophotometric method described by Vaher et al. [24]. A total of 0.2 mL of every fraction of free phenolics was blended with 1 mL of Folin–Ciocalteau reagent and 0.8 mL of a saturated sodium carbonate (Na_2CO_3) solution (7.5%). The prepared mixed solution was stored at room temperature (24 °C) for 30 min in the dark, and, the absorbance was measured at a wavelength of 765 nm with a V-1100D spectrophotometer (J.P. Selecta S.A., Barcelona, Spain). The TPC content was expressed as mg of gallic acid equivalent mL of solution [mg GAE/100 g (DM)] [24]. The ability of the BIB extract to scavenge DPPH· free radicals was assessed using the method described by Zhu et al. [25]. The 400 µl of sample or ethanol (blank) were added to 3600 µl of a 100 µM DPPH ethanolic solution and mixed. Then, after 20 min of storage in the dark at room temperature (24 °C), the absorbance was measured at a wavelength of 517 nm with a V-1100D spectrophotometer (J.P. Selecta S.A., Barcelona, Spain). All measurements were performed in triplicate.

Supplementary File S2_Method for fatty acids

Evaluation of fatty acid (FA) profile in unripened cow milk curd cheese (U-CC)

The extraction of lipids for FA analysis was performed with chloro-form/methanol (2:1 v/v) and fatty acid-methyl esters (FAME) were prepared accord-ing to the method described by Pérez Palacios et al. [27]. FA of the U-CC were identified using a gas chromatograph (GC – 2010 Plus, Shimadzu corp., Kyoto, Japan) equipped with mass spectrometer (MS) (GCMSQP2010, (Shimadzu corp., Kyoto, Japan). Separation was carried out on a Stabilwax-MS column [30 m length, 0.25 mm-internal diameter (ID), and 0.25 µm film thickness (df)] (Restek Corporation, Bellefonte, Pennsylvania, USA). Oven temperature programming started at 50 °C, raised 8 °C / min to 220 °C, held for 1 min at 220 °C, increased again at 20 °C min to 240 °C and, finally, held for the last 10 min. Injector temperature was 240 °C, interface -240 °C and ion source 240 °C. The carrier gas was helium (He) at a flow rate of 0.91 mL/min. Individual FAME peaks were identified by comparing their relative retention times (RRT) with those of standards (Merck & Co., Inc., Kenilworth, New Jersey, USA).

Supplementary File S3_Method for volatile compounds profile

Evaluation of volatile compounds (VC) in unripened cow milk curd cheese (U-CC)

The VCs of U-CC were analyzed by gas chromatography-mass spectrometry (GC-MS) as described by Bartkiene et al. [28] with slight modifications. Solid-phase microextraction (SPME) device with Stableflex (TM) fiber coated with 85 μm polydimethylsiloxane (PDMS)-Carboxen™ layer (Supelco, Bellefonte, Pennsylvania, USA) was used to prepare the samples. For headspace extraction, 2 g of sample in the 20 mL extraction vial sealed with polytetrafluoroethylene (PTFE) septa was thermostated at 60 °C for 15 min, exposing the fiber in the headspace. The fiber was exposed to the headspace of the vial for 10 min. The desorption time was 2 min. Prepared samples were analyzed with a GC-MS-QP2010 (Shimadzu, Kyoto, Japan) gas chromatograph with mass spectrometer. The following method conditions were used for analyses: injector temperature 250 °C, ion source temperature 220 °C, interface temperature 280 °C. Sample injection was carried out for 2 min in order to ensure full desorption of volatiles from the SPME fiber. The temperature gradient was programmed from start at 35 °C (5 min-hold) to 200 °C (10 °C / min) up to 280 °C (25 °C / min) (5 min-hold). The VCs were identified according to the mass spectra libraries (NIST11, NIST11S, FFNSC2).

Supplementary File S4_Method for biogenic amines content

Evaluation of biogenic amine (BA) content in unripened cow milk curd cheese (U-CC)

The extraction and determination of BA in U-CC followed the procedures developed by Ben-Gigirey et. al. [30], with some modifications as described by Bartkiene et al. [31]. The derivatization of sample extracts and standards was performed using a dansyl chloride solution in acetonitrile (10 mg/mL) as a reagent. A Varian ProStar HPLC system (Varian Corp., Palo Alto, CA, USA) equipped with a ProStar 325 UV/VIS Detector and Galaxy software (Agilent, Santa Clara, CA, USA) was used for analysis. A Discovery® HS C18 column (150 × 4.6 mm, 5 μm; SupelcoTM Analytical, Bellefonte, PA, USA) was used to separate BA. Ammonium acetate (0.1 mol/L) and acetonitrile were used as the mobile phases at a flow rate of 0.8 mL/min. The sample volume injected was 20 μL and the amines were monitored at 254 nm. The BA were identified based on their retention times in comparison to their corresponding standards.

Supplementary File S5_Method for evaluation of overall acceptability and induced emotions for consumers

Evaluation of overall acceptability and induced emotions for consumers in unripened cow milk curd cheese (U-CC)

The overall acceptability of the U-CC was established by 10 trained judges, according to International Standards Organization (ISO) method 6658:2017 [32], using a 10-point scale ranging from 0 ("extremely dislike") to 10 ("extremely like"). Ten judges were recruited internally (Institute of Animal Rearing Technologies and Department of Food Safety and Quality, Lithuanian University of Health Sciences, Kaunas, Lithuania): 5 females and 5 males, from 25 to 50 years old [33,34]. Individuals who were familiar with this study were excluded from the panel. The previous training of the judges was based on descriptive analysis [35–37]. Selected judges were non-smokers, interested in sensory analysis and motivated to participate.

In parallel, U-CC samples were tested by applying FaceReader 6.0 software (Noldus Information Technology, Wageningen, The Netherlands), scaling eight emotion patterns (neutral, happy, sad, angry, surprised, scared, disgusted and contempt) [38].

In the measurement experiments, judges were asked to rate the U-CC samples during and after consumption with an intentional facial expression, which was recorded and then characterized by FaceReader 6.0. The judges were asked to taste the whole sample at once, take 15 s to reflect on the taste impressions, then give a signal with a hand and visualize the taste experience of the sample with a facial expression best representing their liking of the sample. For each sample, the section of intentional facial expression (from the exact point at which the subject had finished raising their hand to give the signal until the subject started lowering their hand again) was extracted and used for statistical analysis.
